# The bone-protective mechanisms of active components from TCM drugs in rheumatoid arthritis treatment

**DOI:** 10.3389/fphar.2022.1000865

**Published:** 2022-10-25

**Authors:** Qingyi Lu, Jie Xu, Haixu Jiang, Qiuzhu Wei, Runyue Huang, Guangrui Huang

**Affiliations:** ^1^ School of Life Sciences, Beijing University of Chinese Medicine, Beijing, China; ^2^ School of Chinese Materia, Beijing University of Chinese Medicine, Beijing, China; ^3^ The Second Affiliated Hospital of Guangzhou University of Chinese Medicine (Guangdong Provincial Hospital of Chinese Medicine), Guangzhou, China

**Keywords:** rheumatoid arthritis, traditional Chinese medicine, osteoclast, active components, bone protection, bone destruction

## Abstract

Rheumatoid arthritis (RA) is an autoimmune disease whose hallmarks are synovial inflammation and irreversible bone destruction. Bone resorption resulting from osteoclasts involves the whole immune and bone systems. Breakdown of bone remodeling is attributed to overactive immune cells that produce large quantities of cytokines, upregulated differentiation of osteoclasts with enhanced resorptive activities, suppressed differentiation of osteoblasts, invading fibroblasts and microbiota dysbiosis. Despite the mitigation of inflammation, the existing treatment in Western medicine fails to prevent bone loss during disease progression. Traditional Chinese medicine (TCM) has been used for thousands of years in RA treatment, showing great efficacy in bone preservation. The complex components from the decoctions and prescriptions exhibit various pharmacological activities. This review summarizes the research progress that has been made in terms of the bone-protective effect of some representative compounds from TCM drugs and proposes the substantial mechanisms involved in bone metabolism to provide some clues for future studies. These active components systemically suppress bone destruction *via* inhibiting joint inflammation, osteoclast differentiation, and fibroblast proliferation. Neutrophil, gut microenvironment and microRNA has been proposed as future focus.

## Introduction

Rheumatoid arthritis (RA) is a common autoimmune disease characterized by synovial inflammation and bone destruction. Early diagnosis and appropriate treatment are greatly meaningful for patients with risk factors for poor outcomes, such as high disease activity, the presence of autoantibodies, and early joint damage (Smolen et al., 2016). As one of the clinical indices, bone erosions are often attributed to irregular and ineffective treatment (Schett and Gravallese, 2012). The accompanying joint deformity of RA impairs their capacity for independent living, thus increasing the economic and psychological burden on their families (Ru et al., 2019).

The insidious onset of RA is connected with genetic, epigenetic and environmental factors. Activated immune system cells (macrophages, dendritic cells, neutrophils, B-cells and T-cells), inflammatory mediators (autoantibodies, cytokines, chemokines and proteases) and nonimmune factors (microbiota) are involved in the progression of synovial inflammation and bone destruction (Fang et al., 2020). The inflammatory state of the host stimulates osteoclasts to surpass osteoblasts, causing bone resorption instead of physiological bone remodeling maintained by balanced bone metabolism (Auréal et al., 2020).

The current treatment includes corticosteroids, traditional disease-modifying anti-rheumatic drugs (cDMARDs), nonsteroidal anti-inflammatory drugs (NSAIDs), biologics and alternative medicine, which indicates traditional Chinese medicine (TCM) in China. TCM has been employed to treat rheumatism diseases for thousands of years. The complex prescriptions that have shown significant efficacy in RA are composed of many effective ingredients, the explicit working mechanisms of which are still not understood. To date, many efforts have been made to reveal the potential targets regulated by the active components in RA. In this review, we outline the potential mechanisms whereby the active components from TCM botanical drugs inhibit bone resorption in RA and the latest findings of some representative compounds.

## Potential therapeutic targets of drugs in bone protection in RA

Bone resorption occurring in the joints of RA patients arises from overactivated immune cells, inflammatory effectors, imbalances in bone metabolism, microbiota dysbiosis and other nonimmune factors.

### Osteoclasts and osteoblasts

RA is often characterized by the destruction of juxta-articular bone and erosions due to bone resorption mediated by osteoclasts with elevated activities (Maruotti et al., 2011). Bone resorption attributes to the imbalance of bone metabolism mainly mediated by osteoclasts and osteoblasts. Osteoclasts are the principal cells responsible for bone resorption in RA. The number of osteoclasts increases when they are derived from bone marrow myeloid progenitors, peripheral monocytes and immature dendritic cells (Speziani et al., 2007). With the increased number of osteoclast precursors followed by enhanced activity and prolonged lifespan of osteoclasts, bone metabolism becomes imbalanced, leading to bone resorption and joint dysfunction. Macrophage colony-stimulating factor (M-CSF) and the ligand for the receptor activator of NF-κB (RANKL) are essential in osteoclast formation. M-CSF binds to receptors on osteoclast precursors. RANKL activates the receptor activator of NF-κB (RANK) on the surface of osteoclast precursors and subsequently activates downstream signaling pathways such as mitogen-activated protein kinases (MAPKs) and NF-κB. Finally, nuclear factor of activated T-cell cytoplasmic 1 (NFATc1), the master transcription factor of osteoclast differentiation, is upregulated and induces the expression of osteoclast function-associated genes, especially tartrate acid phosphatase (TRAP) (Takegahara et al., 2022). Costimulatory signals are also involved, such as immunoreceptor tyrosine-based activation motif (ITAM)-associated receptors and Toll-like receptors (TLRs). ITAM contributes to the activation of NFATc1, but the explicit mechanism by which TLRs assist in osteogenesis remains unknown (Auréal et al., 2020).

On the other hand, osteoblasts are devoted to building the host skeleton. They play an essential role in bone formation by synthesizing bone-associated proteins to form extracellular matrix and then mineralize (Wehmeyer et al., 2016). Osteoblasts are derived from mesenchymal stem cells (MSCs) through bone morphogenic protein (BMP) pathways and wingless-related integration site (Wnt) pathways (Ponzetti and Rucci, 2021). Transforming growth factor-β (TGF-β)-involved BMP pathways can be divided into Smad-dependent and non-Smad-dependent pathways, which both upregulate the transcription of runt-related transcription Factor 2 (RUNX2) (Chen et al., 2012). Wnt/β-catenin signaling regulates osteogenesis by repressing alternative differentiation pathways of MSCs, promoting osteoblast differentiation, proliferation, and mineralization activity while blocking osteoblast apoptosis (Ponzetti and Rucci, 2021). In addition, *β*-catenin represses osteoclastogenesis by inducing the expression of osteoprotegerin (OPG), which inhibits RANK-RANKL signal transduction. Dickkopf (DKK) families and Sclerostin are inhibitors of lipoprotein receptor–related protein 5 and 6 (LRP5/6) coreceptors that activate the Wnt pathway in osteoblast differentiation (Krishnan et al., 2006). Dickkopf-related protein 1 (DKK1) is inhibited by TGF-β in the mineralization stage of osteoblast differentiation in osteoprogenitors, which induces alkaline phosphatase (ALP) and collagen synthesis in the extracellular matrix maturation stage (Nam et al., 2020). After bone deposition, osteoblasts go on to apoptosis, or become bone lining cells or osteocytes (Ponzetti and Rucci, 2021). Given the mechanism of bone metabolism, TCM-mediated regulation of the differentiation and activity of osteoblasts and osteoclasts is an important therapeutic target in RA.

### Synoviocytes

Fibroblast-like synoviocytes (FLS) also execute bone and cartilage degradation in RA progression (Harre and Schett, 2017). Under RA conditions, FLS undergo epigenetic changes toward an inflammatory phenotype (Neumann et al., 2010) that highly expresses tumor necrosis factor (TNF), interleukin-6 (IL-6), IL-1β and proteases, which fuel further inflammation and destruction of bone (Nygaard and Firestein, 2020). The production of OPG by FLS can be downregulated by TGF-β through Smad signaling (Hase et al., 2008). Active FLS primarily affect the activity of osteoblasts and osteoclasts. They induce osteoclast differentiation by producing RANKL in inflamed joints (Harre and Schett, 2017) in response to IL-17, IL-6, IL-1β (Hashizume et al., 2008) and autoantibodies (Kurowska et al., 2021). TNF-induced RANKL expression by FLS can be inhibited by sclerostin, which is mainly produced by osteocytes and is thought to suppress osteoblast formation (Wehmeyer et al., 2016; Weivoda et al., 2017). In addition, FLS regulate TNF-stimulated osteoblasts by expressing DKK-1 (Yeremenko et al., 2015). Additionally, migrating proliferated FLS invade and destroy the normal structure of bone. Therefore, it is beneficial for bone protection in RA treatment to consider FLS as promising therapeutic targets (Nygaard and Firestein, 2020).

### Neutrophils

Neutrophils, as an important part of innate immunity, play an important role in the initiation and development of RA. Neutrophils account for over 90% of cells in synovial fluid from RA patients (Weissmann and Korchak, 1984). They participate in the inflammation and bone erosion of RA by releasing TNF, IL-1β, IL-6 and other cytokines, as well as matrix metalloproteinase-9 (MMP-9), myeloperoxidase (MPO) (Strzepa et al., 2017) and neutrophil elastase (NE) (Krotova et al., 2020), which degrade the extracellular matrix to impair joints (Chen et al., 2018). In addition, the observation that neutrophils from RA cases exhibited increased spontaneous NET formation *in vitro* with enhanced NE and MPO expression and PAD-4-mediated citrullination of H3 (Sur Chowdhury et al., 2014) confirmed that NET formation is one of the mechanisms whereby neutrophils transmit immune responses in RA. Peptidyl-arginine-deiminases-4 (PAD-4) has been proven to be indispensable for NET formation and its antibacterial capability (Li et al., 2010). Autophagy is also closely associated with NET formation in that autophagy inducers significantly promote NET formation (Guo et al., 2021), while silencing *Atg5* blocks NET formation (Xu et al., 2017). During the formation of NETs (Brinkmann et al., 2004), the extracellular components of NETs expose citrullinated and carbamylated autoantigens (O'Neil et al., 2020) to dendritic cells and macrophages for antigen presentation, thus activating B-cells for the subsequent synthesis of antibodies and the formation of ICs (O'Neil and Kaplan, 2019). Given that the production of antibodies is closely related to neutrophils, it is essential to inhibit the inflammatory responses and NET formation of neutrophils in the treatment of RA to reduce bone erosion (Wu et al., 2020).

### B-cells

B-cells are responsible for producing osteoclasogenic cytokines such as IL-6 (Mihara et al., 2011) and RANKL (Yeo et al., 2011) in RA progression, although they differentiate into regulatory B-cells that produce IL-10 and TGF-β to suppress osteoclasts (McInnes et al., 2016). Fc-receptor-like-4 (FcRL4) positive B-cells (Yeo et al., 2015), memory B-cells and the regulatory B10 cells were recognized as RANKL-producing subsets (Hu et al., 2017). The pathogenic RANKL-producing B10 cells were positively correlated with disease activity (Hu et al., 2017). More importantly, B-cells form plasma cells that produce autoantibodies in response to the emergence of autoantigens. The presence of autoantibodies such as rheumatoid factor (RF) are often recognized as hallmarks and clinical indices of RA (van Delft and Huizinga, 2020). The anti-citrullinated protein antibody (APCA) is another common and relatively characteristic autoantibody in RA. Citrullinated peptides that can serve as autoantigens are detectable in RA patients (Van Steendam et al., 2010; Sakkas et al., 2014). The neoepitopes may arise from NET formation (Karmakar and Vermeren, 2021). Autoantibodies in the RA can induce osteoclasts by inducing RANKL secretion in FLS (Kurowska et al., 2021). The formation of immune complexes (ICs) also activates the completement system, triggering downstream inflammation to induce local and systemic bone loss (Komatsu and Takayanagi, 2018).

### T-cells

The presentation of autoantigens by dendritic cells leads to the generation of various T helper (Th) cells, including Th1, Th2, Th17 and T follicular helper (Tfh) cells. In particular, the proportion of regulatory T (Treg)/Th17 cells plays a fundamental role in autoimmune diseases. An imbalance between Treg cells and Th17 cells often emerges in RA (Komatsu and Takayanagi, 2018). The transcription factor forkhead Box P3 (Foxp3) is critical for Treg differentiation and suppressive capability (Kondo et al., 2018) and is critically downregulated under RA conditions. Th17 differentiation can be induced by IL-1β and IL-6 through the expression of RAR-related orphan receptor gamma (RORγt) (Chung et al., 2009) and signal transducer and activator of transcription 3 (STAT3) (Nishihara et al., 2007), respectively. The ratio of Treg/Th17 often declines in RA, leading to the accumulation of inflammation with increased concentrations of IL-17, IL-23, IL-6, and TNF-α (Niu et al., 2012). Th1, Th2 and Treg subsets (Kelchtermans et al., 2009) inhibit osteoclastogenesis directly or indirectly through cytokines, including IFN-γ (Tang et al., 2018), IL-10 (Luo et al., 2011), IL-4 (Kim Y. G. et al., 2007), TGF-β(Luo et al., 2011) and cytotoxic T-lymphocyte antigen 4 (CTLA-4) (Axmann et al., 2008). Th17 cells are the primary T-cell subset leading to osteoclastogenesis by synthesizing IL-17 (Sato et al., 2006). Tregs secrete cytokines such as IL-10 and TGF-β to suppress the differentiation of osteoclasts and their resorptive activities (Luo et al., 2011). In view of the threat posed by the imbalance of Treg/Th17 to bone protection of RA, it is imperative to consider regulating the frequencies and activities of Th17 and Treg cells in RA treatment to limit bone destruction.

### Cytokines

The presence of different cytokines in the synovial fluid of RA patients has been studied. TNF-α, IL-1β, IL-6 and IL-17 contribute to osteoclastogenesis. TNF-α is able to reduce osteoblast differentiation by inhibiting the expression of insulin-like growth Factor 1 (IGF-1) (Gilbert et al., 2000) and RUNX2 (Gilbert et al., 2002) in different stages. IL-1β induces MMPs and the differentiation of osteoclasts (Abramson and Amin, 2002) and Th17 cells (Chung et al., 2009). The proinflammatory effect of IL-6 is similar to that of TNF (Tanaka et al., 2014). For osteoclastogenesis, IL-6 directly induces RANKL expression in RA-FLSs and IL-6 is essential for RANKL induction by TNF and IL-17 (Hashizume et al., 2008). In combination with TGF-β, IL-6 is indispensable for Th17 differentiation (Korn et al., 2009). IL-17 activates FLS to produce RANKL (Komatsu and Takayanagi, 2018) and stromal cell-derived Factor 1 (SDF-1) (Kim K. W. et al., 2007). SDF-1 promotes the recruitment of osteoclast precursors, differentiation and bone resorptive activity of osteoclasts (Kim et al., 2014), as well as the expression of MMPs and IL-6 in RA (Villalvilla et al., 2014; Bragg et al., 2019).

Some essential cytokines counteract the osteoclastogenic effect of RANKL. IFN-γ strongly inhibits the fusion of osteoclasts originating from DCs, as well as TRAP and bone resorption activities (Speziani et al., 2007). IL-4 significantly increases OPG secretion by fibroblasts (Tunyogi-Csapo et al., 2008). CTLA-4 induces the apoptosis of osteoclast precursors (Takayanagi, 2015). TGF-β activates osteoblasts through the BMP pathway (Chen et al., 2012) while inhibiting OPG production from FLS in RA (Hase et al., 2008). The interplay between osteoclasts and osteoblasts can be regulated by targeting the network of cytokines and chemokines.

### Microbiota

Organisms distributed in the oral and intestinal cavities contribute to the homeostasis of the host. With the brain-gut axis recognized as the primary effector in many diseases, research on the brain-gut-bone axis is starting to gain attention (Seely et al., 2021). As is seen in osteoporosis (Seely et al., 2021), gut organisms may affect osteoclastogenesis and bone remodeling progression through the regulation of RANKL. The functions that microbiota may perform in the progression of bone destruction in RA have several aspects. First, microbiota is closely related to bone metabolism, but the explicit mechanism is unknown. The implantation of gut bacteria from mice with conventional specific pathogen-free to germ-free mice increases bone mass with a significant increase in IGF-1 in serum (Yan et al., 2016). Supplementation with *Lactobacillus rhamnosus GG* increases trabecular bone volume in mice (Tyagi et al., 2018). In addition, microbiota dysbiosis affects the overall inflammation of the host. They are part of the physical and chemical barriers of the intestine (Mu et al., 2017). Changes in the microbiota constitution influences the expression level of TLRs of antigen-presenting cells and Th subsets (Chen et al., 2017). *Lactobacillus casei* was reported to alleviate bone loss in rats with adjuvant-induced arthritis (AA) by restoring the composition and function of microbiota and inhibiting proinflammatory cytokines (Pan et al., 2019). Furthermore, microbial metabolites, such as short-chain fatty acids (SCFAs), have been shown to play significant roles in the immune system. The protective effects of SCFAs on bone mass are associated with the inhibition of osteoclast differentiation and bone resorption *in vitro* and *in vivo* through the downregulation of NFATc1-involved pathways (Lucas et al., 2018). Metabolites also regulate the differentiation and activity of B-cells and T-cells (Huang et al., 2021). Specifically, butyrate suppresses the differentiation of osteoclasts *in vitro* and promotes Treg subsets *in vivo* (He et al., 2022). It regulates Treg cells to stimulate the differentiation of osteoblasts *via* the upregulation of Wnt10b (Tyagi et al., 2018). Butyrate administration alleviates joint swelling and increases the frequency of Treg cells and the concentration of IL-10. Butyrate also decreases the frequency of Th17 cells *in vivo* (Hui et al., 2019). Considering the function of the microbiome, modulating aberrant species and metabolites is one of the promising therapeutic targets of natural compounds in RA bone protection.

### Other effectors

Apart from the immune cells discussed above, macrophages (Cutolo et al., 2022) and dendritic cells are responsible for antigen presentation, as is the synthesis of related cytokines in the inflammatory response. Some novel mechanisms whereby osteoclast differentiation is regulated have been proposed, such as noncoding RNA (ncRNA)-involved pathways (Ponzetti and Rucci, 2021). However, research on the effect of active components from TCM on bone protection in RA is somewhat limited to the classic mechanism.

**FIGURE 1:**
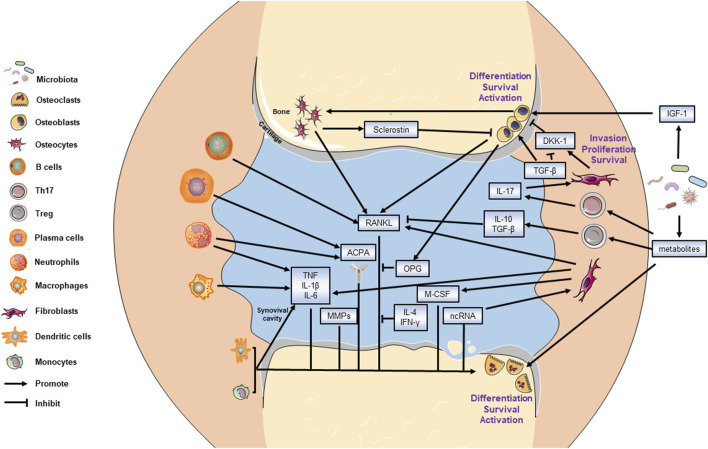
Mechanism of bone metabolism in RA. All targeted cells and cytokines are labeled. The bone resorptive activity is balanced by osteoblasts and osteoclasts. The invasion of fibroblasts also leads to bone destruction. The arrow end represents promotion effect while the blunt end represents the inhibitory effect.

Collectively, the interplay between bone metabolism and immunology in RA is delineated in Figure 1.

## Representative effective components of TCM botanical drugs in the treatment of RA

**FIGURE 2:**
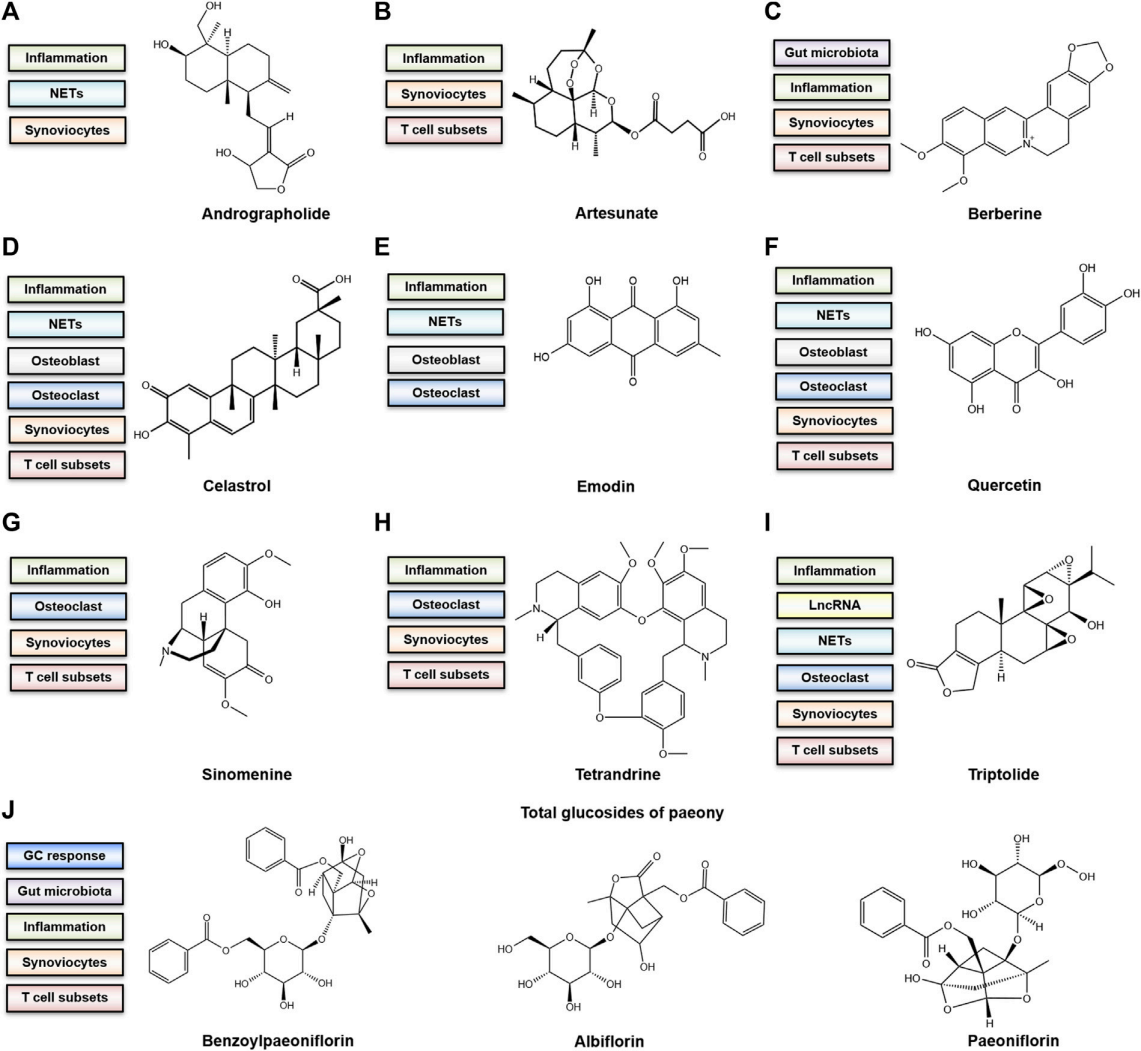
The bone-protective mechanism and chemical structure of active components **(A)** Andrographolide. **(B)** Artesunate **(C)** Berberine. **(D)** Celastrol **(E)** Emodin. **(F)** Quercetin **(G)** Sinomenine. **(H)** Tetrandrine **(I)** Triptolide. **(J)** Total glucosides of paeony (TGP). The chemical structure of representative ingredients from TGP are shown.

The current treatment strategies for RA have some shortcomings despite satisfactory efficacy in the alleviation of symptoms and progression (Emery, 2006). Long-term administration of immunosuppressants and the excessive use of corticosteroids often result in serious adverse reactions (Bresalier et al., 2005; Sardar and Andersson, 2016; Smolen et al., 2020). The high expenses, unexpected side effects and low response of some patients to biologics make it difficult to promote biologics extensively (Keyser, 2011). In China, thousands of years have witnessed the efficacy of TCM in RA treatment. A systematic review overlooking the bone-protecting efficacy of TCM alone or in combination with Western medicine in the treatment of RA has proposed that Chinese decoctions have advantages over Western medicine in terms of bone protection (Shi et al., 2020). Since it is difficult to explore the enigmatic mechanism of the complex prescriptions, the functions of each single component of TCM botanical drugs are worth investigating. Notably, evaluating the molecular targets of some natural components showing great efficacy in RA can assist in improving the understanding of key underlying molecular mechanisms (Shen et al., 2020). Here, we provide an overview of recent research advances in TCM in the frontiers of RA osteoclastogenesis. The representative bioactive compounds that have been proven to be bone protective in animal models of RA will be primarily discussed. The main mechanism of the representative components and their chemical structure is depicted in Figure 2. The cells that are targeted by these compounds both *in vivo* and *in vitro* along with the adopted animal models are listed in Table 1.

**TABLE 1:** Targeted cells and animal models used in the studies of active components from TCM.

Compound	Deteced cells and cell lines	Animal models
Andrographolide	FLS Yan et al. (2012); Li et al. (2017); Macrophage Gupta et al. (2018); Neutrophil Li et al. (2019b); Luo et al. (2020)	AA Gupta et al. (2018); Li et al. (2019b); Luo et al. (2020); CIA Li et al. (2017)
Artesunate	Treg Liu et al. (2017); Th 17 Liu et al. (2017); FLS Xu et al. (2007); Zhu et al. (2016); Ma et al. (2019)	CIA Liu et al. (2017)
Berberine	Th17 Yue et al. (2017); Dinesh and Rasool, (2019); Tfh cell Vita et al. (2021); Treg Dinesh and Rasool, (2019); Vita et al. (2021); FLS Dinesh and Rasool, (2019)	CIA Wang et al. (2014); Yue et al. (2017); Yue et al. (2019); Vita et al. (2021)
Celastrol	RAW 264.7 Nanjundaiah et al. (2012); Gan et al. (2015); SIC Nanjundaiah et al. (2012); Astry et al. (2015); MC3T3-E1 Nanjundaiah et al. (2012); FLS Xu et al. (2013); Fang et al. (2017); Th17 Astry et al. (2015); Treg Astry et al. (2015); Neutrophil Yu et al. (2015); THP-1 Jing et al. (2021)	CIA Gan et al. (2015); Gao et al. (2020); AA Nanjundaiah et al. (2012); Astry et al. (2015); Jing et al. (2021)
Emodin	BMDMs Hwang et al. (2013); MC3T3-E1 Lee et al. (2008); Neutrophil Zhu et al. (2019)	CIA Hwang et al. (2013); AA Zhu et al. (2019)
Quercetin	MC3T3-E1 Yamaguchi and Weitzmann, (2011); RAW264.7 Yamaguchi and Weitzmann, (2011); FLS Kim et al. (2019); Treg Yang et al. (2018); Kim et al. (2019); Th17 Yang et al. (2018); Kim et al. (2019); PBMCs Kim et al. (2019); Neutrophil Yuan et al. (2020)	CIA Yang et al. (2018); AA Yuan et al. (2020)
Sinomenine	RAW 264.7 He et al. (2014); FLS Yi et al. (2018); Treg Tong et al. (2015); Th17 Tong et al. (2015); PBMCs Tong et al. (2015); Xu et al. (2021)	AA Mu et al. (2013); CIA Tong et al. (2015)
Tetrandrine	RAW 264.7 Jia et al. (2018); Jia et al. (2019); BMDMs Jia et al. (2018); Jia et al. (2019); EL-4 Yuan et al. (2016); Th17 Yuan et al. (2016); Yuan et al. (2017); Treg Yuan et al. (2016); MH7A Lv et al. (2015); FLS Lv et al. (2015); Neutrophil Lu et al. (2022)	CIAYuan et al. (2016); Jia et al. (2018); AA Lu et al. (2022)
Triptolide	FLS Liu et al. (2013); Yang et al. (2016); Piao et al. (2021); PBMCs Liu et al. (2013); Treg Xu et al. (2016); BMDMs Xu et al. (2016); Th17 Shen et al. (2022); Neutrophil Huang et al. (2018)	CIA Liu et al. (2013); Yang et al. (2016); Piao et al. (2021); Shen et al. (2022); SCID with coimplantation Yang et al. (2016); AA Huang et al. (2018)
Total glucosides of paeony	GC B cells Li et al. (2019a); Tfh cells Li et al. (2019a); FLS Jia et al. (2014); PBMCs Peng et al. (2019); Th1 Peng et al. (2019); Th2 Peng et al. (2019); Th17 Peng et al. (2019); Treg Peng et al. (2019)	CIA Jia et al. (2014); Li et al. (2019a); Peng et al. (2019); AA Wei et al. (2013)

### Andrographolide

Andrographolide (Figure 2A) is the major diterpenoid bioactive compound derived from *Andrographis paniculata* (Burm.f.) Nees [Acanthaceae]*.* The X-ray examination showed that the oral administration of andrographolide (25, 50 and 100 mg/kg) has a bone-protective effect in AA rats (Luo et al., 2020). It reduced the expression of TNF-α and IL-6 in the serum (Luo et al., 2020). *In vivo*, the upregulation of antioxidant enzymes such as superoxide dismutase and the downregulation of CXCL2, elastase and myeloperoxidases certainly contribute to the alleviation of oxidative stress measured by malondialdehyde, catalase, glutathione, superoxide dismutase and the ratio of nitrite/nitrate (Luo et al., 2020). The joint symptoms of the collagen-induced arthritis (CIA) mice treated with daily andrographolide (100 mg/kg) orally were alleviated. Also, andrographolide reduced serum anti-collagen II antibody, TNF-α, IL-1β and IL-6. Andrographolide (10 or 20 μM) showed a suppressive effect on TNF-α activated FLS by blocking the phosphorylation of P38 and ERK *in vitro* (Li et al., 2017). In AA rats, andrographolide (50 mg/kg) was given intraperitoneally (Gupta et al., 2018). Andrographolide (0.5 μg/ml) decreased LPS induced overexpression of COX-2, iNOS and NF-κB p65 in macrophages *in vitro*, although the reductions were insignificant (Gupta et al., 2018). Andrographolide (10–30 μM) further inhibited proliferation of FLS isolated from RA patients by arresting the cell cycle at the G0/G1 stage and triggering apoptosis *in vitro* (Yan et al., 2012). Reducing NET formation was also effective in ameliorating RA by andrographolide (Li X. et al., 2019). The oral administration of andrographolide (25–50 mg/kg) greatly ameliorated joint swelling of AA mice (Li X. et al., 2019). The local infiltration of neutrophil and formation of NETs was also inhibited *in vivo* (Li X. et al., 2019). *In vitro,* the treatment of andrographolide (25 μM) balanced autophagy and NET formation (Li X. et al., 2019). Additionally, although the changes in microbiota induced by andrographolide in animal models with RA remain undetermined, beneficial variation in the microbiota composition in blank mice treated with andrographolide (2–20 mg/kg) points to the hypothesis that regulation of gut microorganisms is one of the mechanisms whereby andrographolide ameliorates bone destruction (Wu et al., 2021).

### Artesunate

Artesunate (Figure 2B), a derivative of artemisinin from *artemisia annua* L [Asteraceae], has been an effective antioxidant. As molybdenum target X-rays showed, oral administration of artesunate (5, 10 or 20 mg/kg) attenuated bone destruction in CIA rats (Liu et al., 2017). The expression of Foxp3 and IL-17 in the synovium and T-cells was also regulated by artesunate *in vivo*, which reflected the rebalance of Th 17 and Treg cells (Liu et al., 2017). *In vitro* assays indicated that artesunate (5, 10 or 20 ng/ml) also modulated Foxp3 expression of synovial cells isolated from CIA rats (Zhu et al., 2016). As for FLS, artesunate (10 μM) regulated the activation of FLS from RA patients by inhibiting the phosphorylation of protein kinase B (PKB) and decreased the secretion of IL-1β and IL-6 *in vitro* (Xu et al., 2007). Artesunate treatment (60 μM) also suppressed the horizontal and vertical migration of FLS from RA patients *in vitro* through 3-phosphoinositide-dependent protein kinase 1 (PDK-1) pathway, as well as the inhibition of MMP-2 and MMP-9 production (Ma et al., 2019). The above findings have revealed that artesunate may affect bone resorption in RA in an immunoregulatory way. Further research focusing on the effect of artesunate on the activity of osteoclast and osteoblast *in vivo* is worth performing.

### Berberine

Berberine (Figure 2C) is a bioactive isoquinoline alkaloid compound from *Coptis deltoidea* C.Y.Cheng and P.K.Hsiao [Ranunculaceae] that has been applied in the treatment of bone diseases for thousands of years known as Huanglian in China. Studies have defined its function as promoting bone regeneration and anti-inflammation (Zhang et al., 2021). The X-ray scanning confirmed that oral administration of berberine (200 mg/kg) protected bone from erosions in CIA rats with decreased transcription levels of TNF-α, IL-1β, IL-6 and RANKL in the synovium, as well as reduced Th17 differentiation by attenuating the phosphorylation of STAT3 in the spleen (Yue et al., 2017). Berberine also induced the production of cortistatin whose receptor antagonists reversed the anti-arthritic efficacy of berberine *in vivo* (Yue et al., 2017). In CIA rats, the treatment with berberine orally (200 mg/kg) reduced the expression levels of TNF-α, IL-1β, IL-6 and IL-17 in sera partly through the MAPK signaling pathway (Wang et al., 2014). In the *in vitro* assays, the FLS and T-cells were purified from AA rats. Berberine (15–45 μM) inhibited the IL-21-induced autophagy of FLS through phosphoinositide three kinase (PI3K)/AKT pathway in a dose dependent manner (Dinesh and Rasool, 2019). It also recovered the balance of Treg/Th17 by increasing the differentiation of Treg cells and reducing the IL-21-induced differentiation of Th17 cells *via* downregulation of RORγt (Dinesh and Rasool, 2019). Another *in vivo* study confirmed the upregulation of Treg cells and downregulation of Tfh cells, as well as the reduction of anti-bovine type II collagen in CIA mice after berberine treatment intraperitoneally (1 mg/kg) (Vita et al., 2021). Interestingly, oral administration of berberine (200 mg/kg) has been shown to regulate butyrate metabolism by elevating the abundance of butyrate-producing bacteria in CIA rats, thereby limiting the generation of nitrate and stabilizing physiological hypoxia in the intestine (Yue et al., 2019). As mentioned above, butyrate is able to suppress osteoclasts, promote Treg cells (He et al., 2022) and promote Th17 differentiation (Hui et al., 2019). The systematic regulation of berberine is somewhat linked to microbial metabolism.

### Celastrol

Celastrol (Figure 2D), a main constituent from *Tripterygium wilfordii* Hook. f [Celastraceae], has shown anti-inflammatory and bone-protective activities both *in vivo* and *in vitro*. Celastrol exerts a bone-protective effect primarily by inhibiting osteoclast differentiation and activities. In CIA mice, celastrol treatment (3 mg/kg) inhibited the expression of osteoclast-specific genes and transcription factors in the synovium, such as TRAP and NFATc1, supporting the observation of microcomputed tomography (micro-CT) that celastrol protected ankle joints from severe bone erosion *in vivo* (Gan et al., 2015). The mechanism was further explored with RAW 264.7 cells, where celastrol treatment (0.03, 0.1 or 0.3 μM) inhibited RANKL-induced expression of TRAP, c-Fos, c-Jun and NFATc1 through the NF-κB and MAPK pathways *in vitro* (Gan et al., 2015). In heat-killed *Mycobacterium tuberculosis* H37Ra (Mtb)-induced arthritic (AA) rats, celastrol treatment (1 mg/kg) reduced bone resorption significantly. TRAP-positive staining, bone histomorphometry and radiographs of limbs indicated a decrease in osteoclasts and preservation of joint integrity by celastrol (Nanjundaiah et al., 2012). In the synovium-infiltrating cells (SICs) isolated from AA rats treated with celastrol, *in vitro* Mtb sonication stimulation failed to raise the ratio of RANKL/OPG and maintained the level of OPN, IGF-1 and MMP-9 at a lower level than those isolated from untreated AA rats (Nanjundaiah et al., 2012). In IL-17-activated MC3T3-E1 cells, there was also a decline in the ratio of RANKL/OPG, M-CSF and IL-6 regulated by celastrol (0.1 or 0.3 μM) *in vitro* (Nanjundaiah et al., 2012). Celastrol treatment (0.1 or 0.3 μM) also decreased the secretion of MMP-9 in RAW 264.7 in response to RANKL *in vitro* (Nanjundaiah et al., 2012). In addition, celastrol treatment (1, 2 or 5 μM) downregulated the proliferation of FLS by inducing DNA damage, cell cycle arrest, and apoptosis *in vitro* (Xu et al., 2013). One high-throughput analysis on the gene differences of FLS from RA patients with or without celastrol treatment (1 μM) revealed that the expression of several chemokine genes from FLS was downregulated by celastrol (Fang et al., 2017). *In vitro* experiments further indicated that NF-κB p65 pathway was also involved in the celastrol (1 μM)-suppressed IL-6 and MMP-9 production in FLS (Fang et al., 2017). In terms of the Th17/Treg balance, intraperitoneal injection of celastrol (1 mg/kg) reduced the ratio of Th17 to Treg cells in the synovial tissue (Astry et al., 2015). In the *in vitro* culture of mice T-cells, celastrol treatment (0.1–0.3 μM) inhibited Th17 differentiation through STAT3 (Astry et al., 2015). Additionally, celastrol treatment (5 or 10 μM) inhibited the neutrophil oxidative burst and NET formation induced by TNFα, ovalbumin:anti-ovalbumin immune complexes (Ova ICs) and immunoglobulin G (IgG) purified from the sera of RA patients (Yu et al., 2015). Intraperitoneal injection of celastrol (1 mg/kg) has shown anti-inflammatory effect on CIA rats *in vivo*, which was measured by the decreased TNF-α, IL-1β, IL-6 and oxidative stress. The effect was abolished by the injection of recombinant adenoviral vectors harboring NADPH oxidase in CIA rats (Gao et al., 2020). The decline of IL-1β and IL-18 in AA rats may be related to the inhibitory effect of celastrol (0.5 or 1 mg/kg) on ROS-NF-κB-NLRP3 (Jing et al., 2021). In LPS-induced human mononuclear macrophages (THP-1 cells), celastrol (12.5–50 nM) suppressed ROS-NF-κB-NLRP3 activation *in vitro* (Jing et al., 2021). Collectively, celastrol inhibits bone resorption in RA treatment in a multitarget way.

### Emodin

Emodin (Figure 2E) is an active ingredient in some Chinese botanical drugs, such as *Rheum palmatum* L [Polygonaceae]. It has shown antioxidant, anti-inflammatory and immunosuppressive effects in various diseases (Zheng et al., 2021). As illustrated by X-ray and radiological scores, the intraperitoneal injection of emodin (10 mg/kg) into CIA mice alleviated joint swelling with reduced bone destruction. It inhibited the activation of NF-κB pathways and differentiation of osteoclasts *in vivo*, as well as the expression levels of TNF-α, IL-1β, IL-17, RANKL and MMP-1/3 (Hwang et al., 2013). The osteoclast differentiation assay suggested that emodin treatment (10 or 20 μM) inhibited TRAP activity induced by RANKL and M-CSF *in vitro* (Hwang et al., 2013). Another *in vitro* report revealed that in MC3T3-E1 cells, emodin treatment (5 or 10 μM) increased the transcription level of BMP-2 and expression level of ALP through PI3K/AKT/MAPK pathways (Lee et al., 2008). In our former reports, AA mice intraperitoneally injected with emodin (30 μg/kg) showed decreased level of TNF-α, IL-6 and IFN-γ in the serum, which suggested the anti-inflammatory effect of emodin as a whole *in vivo*. In addition, emodin treatment (20 μM) significantly reduced phorbol 12-myristate 13-acetate (PMA)-triggered NET formation *in vitro* (Zhu et al., 2019). In summary, emodin has a regulatory effect on the differentiation of osteoblasts, osteoclasts and T-cells, in addition to its anti-inflammatory and anti-NET functions in RA treatment.

### Quercetin

Quercetin (Figure 2F) is a representative flavonoid that is found in *Morus alba* L [Moraceae]. It is also present in fruits and vegetables. Apart from its anti-inflammatory and other diverse pharmacological activities (Guan et al., 2021), its bone-protective effect has been studied extensively in bone-related diseases such as osteoporosis (Wong et al., 2020). Quercetin performs dual-target regulation of bone metabolism. Quercetin treatment (50 μM) inhibited BMP-2- and TGF-β-induced Smad activation in MC3T3-E1 cells, which led to suppression of mineralization (Yamaguchi and Weitzmann, 2011). For osteoclastogenesis, quercetin suppressed osteoclast differentiation from RAW264.7 elicited by TNF and RANKL in a dose-dependent manner (0.1–25 μM) (Yamaguchi and Weitzmann, 2011). Similar conclusion could be drawn from the *in vitro* results that quercetin (25 μM) inhibited IL-17-stimulated RANKL production in RA-FLS and quercetin (1–25 μM) inhibited RANKL-stimulated TRAP expression in PBMCs (Kim et al., 2019). In the coculture of PBMCs and RA-FLS, quercetin (25 μM) also suppressed osteoclast formation induced by IL-17 (Kim et al., 2019). Interestingly, quercetin (25 μM) showed no effect on Treg differentiation, while it significantly reduced Th17 differentiation and IL-17 production *in vitro* (Kim et al., 2019). In CIA rats, the percentage of Th17 cells increased, and the percentage of Treg cells decreased after oral treatment with quercetin (150 mg/kg) (Yang et al., 2018). The further assays revealed that quercetin inhibited NLRP3 inflammation in the synovial tissues and production of anti-CII IgG2a in the serum (Yang et al., 2018). In AA mice, quercetin administration alleviated joint swelling and reduced NET formation *in vivo*. *In vitro* assays indicated that quercetin (25 μM) probably inhibited NET formation *via* autophagy suppression*.* It was also proposed that quercetin (25 μM) reversed the delay of neutrophil apoptosis induced by LPS *in vitro* (Yuan et al., 2020).

### Sinomenine

Sinomenine (Figure 2G), a major component of *sinomenium acutum* (Thunb.) Rehder and E.H.Wilson [Menispermaceae], has various pharmacological activities, especially a regulatory effect on bone protection in the treatment of RA. In sinomenine-treated AA rats, radiographs indicated that the oral administration of sinomenine (100 mg/kg) had significant protective effect on the joints (Mu et al., 2013). The declined expression levels of MyD88, TLR2, TLR4, TNF-α, IL-1β, and IL-6 in synovial tissues indicated that sinomenine greatly alleviated local inflammation *in vivo* (Mu et al., 2013). In addition, sinomenine treatment (0.25–1 mM) was proven to induce apoptosis of RAW 264.7-derived osteoclasts *in vitro* by activating caspase-3 and disrupting the actin ring structure (He et al., 2014). In terms of FLS, sinomenine (0.25–0.4 mM) further reduced TNF-α induced proliferation of FLS from the synovium of AA rats by inhibiting the expression of alpha seven nicotinic acetylcholine receptors *in vitro* (Yi et al., 2018). The frequency change of Th17 cells and Treg cells in the gut lymphoid tissues of CIA rats was attributed to the oral administration of sinomenine (120 mg/kg) (Tong et al., 2015). Noticeably, the frequency change of Th17 cells and Treg cells in the spleen was insignificant compared with the that of CIA rats (Tong et al., 2015). Sinomenine intervention also enhanced the migration of Treg cells from gut to joint marked by higher expression of Foxp3 in joint tissues (Tong et al., 2015). Decreased IL-17 and increased IL-10 in the serum was observed accordingly (Tong et al., 2015). *In vitro*, sinomenine treatment (1 mM) suppressed the proliferation and secretion of TNF-α and IL-17 in PBMCs activated by type II collagen (CII) (Tong et al., 2015). Interestingly, sinomenine (0.3–30 μM) has been defined as having a limited effect on Th cells and Treg cells from mitogen-activated PBMCs isolated from RA patients *in vitro* (Xu et al., 2021). As previously reported, intraperitoneal injection of sinomenine (20 mg/kg) failed to achieve expected efficacy in CIA rats (Tong et al., 2015). We proposed that the regulatory function of sinomenine may be further related to gut digestion and even the gut microbiota.

### Tetrandrine

Tetrandrine (Figure 2H), known as a bioactive alkaloid derived from the dry root of *stephania tetrandra* S. Moore [Menispermaceae], has been studied in RA research. Yue Dai’s group revealed that tetrandrine attenuated osteoclastogenesis in CIA rats by delineating increased bone mineral density (BMD) and trabecular bone (Tb) of bone parameters in the micro-CT and decreased TRAP expression *in vivo* after oral administration of tetrandrine (30 mg/kg). *In vitro*, tetrandrine treatment (0.1–0.3 μM) greatly inhibited TRAP activity and other osteoclast related genes in both bone marrow-derived macrophages (BMDMs) and RAW264.7 cells. For RAW264.7, this may be related to blocking of the nuclear translocation of NF-κB-p65 and NFATc1 by reducing the activation of spleen tyrosine kinase (Syk) (Jia et al., 2018). Further *in vitro* experiments in RAW 264.7 cells and BMDMs suggested that tetrandrine (0.3 μM) enhanced the ubiquitination and degradation of Syk and downregulated the expression of NFATc1 in an AhR-dependent manner (Jia et al., 2019). The differentiation of T-cell subsets is also regulated by tetrandrine. It was proposed that the oral treatment of tetrandrine (20 or 40 mg/kg) can relieve cartilage destruction and joint swelling by restoring the balance of Th17 and Treg cells in mesenteric lymph nodes with a decrease in TNF-α, IL-1β, IL-6, IL-17A, total IgG and isotype-specific IgG2a in serum from CIA mice. After tetrandrine treatment, IL-10 also rose in CIA mice (Yuan et al., 2016). Then it was observed that tetrandrine (0.3–1 μM) modulated T-cell differentiation through AhR in a series of cell lines (Yuan et al., 2016). The former observation was further explored through elucidating the regulation of STAT-3 and STAT-5 by tetrandrine (1 μM) in inhibiting Th17 differentiation *in vitro* (Yuan et al., 2017). Tetrandrine affects the proliferation and migration of FLS to protect bone in RA. In both primary FLS isolated from the synovium of RA patients and the cell line MH7A, tetrandrine (0.3–1 μM) greatly impeded the migration and invasion of RA-FLS *in vitro*, as well as the expression of MMP-2/9 (Lv et al., 2015). In addition, NET formation was proven to be inhibited in AA mice after tetrandrine treatment with intraperitoneal injection (6 mg/kg) (Lu et al., 2022). *In vitro*, delineation of the reduced expression of PAD-4 and citrullinated histone H3 after tetrandrine treatment (10 μM) supported former observation *in vivo* (Lu et al., 2022).

### Triptolide

Triptolide (Figure 2I) is another extract from the herb *tripterygium wilfordii* Hook. f [Celastraceae]. It alleviates bone destruction in RA in various ways (Fan et al., 2018). As discussed before, bone resorption mediated by osteoclasts mainly gives rise to bone destruction in RA. In CIA mice, micro-CT images suggested a protective function of triptolide in terms of preserved bone volume and quality after oral administration of triptolide (8–32 μg/kg) (Liu et al., 2013). *In vivo* triptolide treatment decreased TRAP-positive cells by downregulating RANKL and RANK along with the upregulation of OPG (Liu et al., 2013). A similar tendency was observed in the coculture of PBMCs and FLS, which supported that RANKL/RANK/OPG signaling was primarily modulated by triptolide in the bone protection of RA (Liu et al., 2013). The *in vitro* coculture of Treg cells and BMDMs with triptolide treatment (10 nM) revealed that triptolide upregulated IL-10 and TGF-β, inhibited the differentiation of osteoclasts, and reduced bone resorptive activities (Xu et al., 2016). The oral administration of triptolide (60 μg/kg) greatly reduced the percentage of Th17 cells in the spleen and the expression of pyruvate kinase M2 (PKM2) compared with that of CIA mice (Shen et al., 2022). Triptolide treatment (0.02–0.08 μg/ml) significantly suppressed Th17 differentiation, IL-17A production and PKM2-mediated glycolysis *in vitro* (Shen et al., 2022). Triptolide further acts on FLS to minimize their invading effect on bone. The migration of FLS and expression of MMP-9 was suppressed in severe combined immunodeficiency (SCID) mice with coimplantation RA model after treatment with triptolide (100 μg/kg) intraperitoneally (Yang et al., 2016). Intraperitoneal injection of triptolide (100 μg/kg) alleviated bone destruction of CIA mice through inhibiting phosphorylation of JNK *in vivo* (Yang et al., 2016). Triptolide (50 nM) inhibited FLS migration and cytoskeleton reorganization *in vitro* (Yang et al., 2016). Regarding ncRNA, triptolide-mediated downregulation of long noncoding RNA (lncRNA) RP11-83J16.1 decreased the proliferation and invasion of FLS in CIA rats after oral administration of triptolide (45 μg/kg) (Piao et al., 2021). So was *in vitro* after triptolide treatment (32 nM) in FLS isolated from RA patients (Piao et al., 2021). In addition to the decreased expression of MPO and NE in AA mice joint tissues after intraperitoneal injection of triptolide (45 μg/kg), *in vitro* treatment of triptolide (200 nM) also inhibited the migration and NET formation of neutrophils (Huang et al., 2018).

### Total glucosides of paeony

Total glucosides of paeony (TGP) from *paeonia lactiflora* Pall [Paeoniaceae] include a series of bioactive ingredients (Figure 2J). They have been proved effective in RA both clinically and experimentally. As micro-CT examinations showed, the bone resorption and joint destruction of the CIA mice treated with TGP intragastrically (0.36 and 0.72 g/kg) was significantly reduced compared with CIA model mice (Li H. et al., 2019). TGP treatment also alleviated inflammation measured by the decreased concentration of anti-CII IgG2a, TNF-α, IL-21 and IL-6 in the serum and reduced phosphorylation of p65 and STAT3 in the paws (Li H. et al., 2019). TGP also exerted immunosuppressive effect on CIA mice through decreasing the number of germinal center B cells and Tfh cells in the spleen (Li H. et al., 2019). The oral treatment of TGP (60 mg/kg) even prevented juxta-articular bone loss in AA rabbits with decreased expression level of RANKL. The FLS proliferation was greatly reduced in CIA rats after oral administration of TGP (25, 50 and 100 mg/kg). *In vitro* assays confirmed that TGP treatment (12.5 or 62.5 μg/ml) inhibited the proliferation of IL-1β-treated FLS from CIA rats, as well as the expression of G proteins (Jia et al., 2014). The oral administration of TGP (158, 474 and 948 mg/kg) lasting for 12 weeks significantly repaired the dysbiosis and dysfunction of the gut microbiota in CIA rats and regulated immune responses in various ways (Peng et al., 2019). TGP treatment also modulated the T-cell subsets in PBMCs and immune responses of the intestinal mucosa from CIA rats. In detail, TGP reversed the imbalance in both Th1/Th2 and Th17/Treg, and regulated the secretion of secretory immunoglobulin A (SIgA) and IFN-γ *in vivo* (Peng et al., 2019).

As shown in Table 1, several active components from TCM that have shown great protective effects on bone in RA modulated multiple cells both *in vivo* and *in vitro* to ameliorate the osteoclastogenic impact. Osteoclasts, osteoblasts, Th cells and FLS play fundamental roles in regulatory mechanisms. In addition, an extensive screen for the effective ingredients from decoctions or botanical drugs that have been clinically used has been performed by Chinese scientists. Hopefully, more components from TCM botanical herbs can be explored and transformed into commercial applications.

## Conclusion

This review has reviewed the existing mechanisms of bone resorption in RA that can be regulated by TCM drugs. Neutrophils, macrophages, B-cells and T-cells participate in inflammatory responses. Along with cytokines, chemokines and proteases, an increase in RANKL stimulates the differentiation of osteoclasts. The weakened differentiation of osteoblasts fails to balance bone resorptive activities. Notably, the microbiota is specifically found to be a promising target for TCM intervention in RA because the gut-joint axis may explain the multitargeted regulation of compounds from TCM. Although numerous active ingredients of TCM prescriptions are effective in the experiments, no single component had achieved ideal effect clinically. Combination of different active components under the direction of TCM theories is a promising strategy to develop new drugs.
